# Exosomal hsa_circ_0006859 is a potential biomarker for postmenopausal osteoporosis and enhances adipogenic versus osteogenic differentiation in human bone marrow mesenchymal stem cells by sponging miR-431-5p

**DOI:** 10.1186/s13287-021-02214-y

**Published:** 2021-03-01

**Authors:** Feng Zhi, Yi Ding, Rong Wang, Yujiao Yang, Kaiming Luo, Fei Hua

**Affiliations:** 1 Department of Neurosurgery, Third Affiliated Hospital of Soochow University, Changzhou City, 213003 Jiangsu China; 2 Department of Geriatrics, Third Affiliated Hospital of Soochow University, Changzhou City, 213003 Jiangsu China; 3 Department of Endocrinology, Third Affiliated Hospital of Soochow University, Changzhou City, 213003 Jiangsu China

**Keywords:** hsa_circ_0006859, Osteoporosis, Exosome, Osteogenesis, Adipogenesis, Human bone marrow mesenchymal stem cells (hBMSCs)

## Abstract

**Background:**

As one of the most common chronic diseases in the world, osteoporosis occurs especially in postmenopausal women. Circular RNAs (circRNAs) are emerging as major drivers in human disease. The aim of the present study was to analyse circRNA expression profiles in osteoporosis and to explore the clinical significance and the regulatory molecular mechanism of hsa_circ_0006859 during osteoporosis.

**Methods:**

Exosomes were isolated from clinically collected serum samples. A circRNA microarray was performed to screen differentially expressed circRNAs. Quantitative real-time PCR (qRT-PCR) and western blot were performed to analyse target gene mRNA expression and protein expression. Alizarin red staining (ARS) was performed to evaluate the mineralization ability of human bone marrow mesenchymal stem cells (hBMSCs). Oil Red O staining was performed to evaluate the lipid droplet formation ability of hBMSCs. Bioinformatics analysis and the luciferase reporter assay were performed to investigate the interaction between two genes.

**Results:**

Hsa_circ_0006859 was identified as one of the most upregulated circRNAs in the microarray analysis. Hsa_circ_0006859 in exosomes was upregulated in osteoporosis patients compared to healthy controls. Hsa_circ_0006859 differentiated osteopenia or osteoporosis patients from healthy controls with high sensitivity and specificity. Hsa_circ_0006859 suppressed osteoblastic differentiation and promoted adipogenic differentiation of hBMSCs. Hsa_circ_0006859 directly bound to miR-431-5p, and ROCK1 was identified as a novel target gene of miR-431-5p. Hsa_circ_0006859 is a competing endogenous RNA (ceRNA) of miR-431-5p that promotes ROCK1 expression. Hsa_circ_0006859 suppressed osteogenesis and promoted adipogenesis by sponging miR-431-5p to upregulate ROCK1.

**Conclusions:**

Exosomal hsa_circ_0006859 is a potential biomarker for postmenopausal osteoporosis and controls the balance between osteogenesis and adipogenesis in hBMSCs by sponging miR-431-5p.

**Supplementary Information:**

The online version contains supplementary material available at 10.1186/s13287-021-02214-y.

## Background

Osteoporosis is a prevalent age-related disease characterized by bone mass reduction and tissue microstructure degeneration, which lead to a high risk of bone fracture, especially in postmenopausal women [1]. Osteoporosis is widely believed to be the consequence of an imbalance between bone formation, which is regulated by osteoblasts, and bone resorption, which is regulated by osteoclasts [2]. Though osteoporosis management strategies have made some strides in the past few years, the complex molecular mechanisms causing osteoporosis and the lack of potential therapeutic targets hinder improvements in its prevention and treatment [3]. Thus, exploring novel targets for the treatment of osteoporosis is imperatively needed.

Circular RNAs (circRNAs) are a novel class of endogenous covalently linked RNA molecules without 5′ to 3′ polarity or a poly A tail [4]. CircRNAs are evolutionarily conserved and are abundant in eukaryotes, suggesting their importance in various physiological conditions [5]. CircRNAs are stable and enriched in exosomes, indicating the potential of circRNAs as promising biomarkers for complex diseases [6, 7]. CircRNAs have multiple functions, such as modulating gene transcription, regulating alternative splicing, modulating translation, interacting with RNA-binding proteins (RBPs), and functioning as microRNA (miRNA) sponges [5]. MicroRNAs (miRNAs) are evolutionarily conserved small non-coding RNAs approximately 22 nucleotides long that inhibit gene expression by base-pairing with the 3′-untranslated region (3′-UTR) or 5′-UTR of mRNAs [8]. Due to the special circular structure, circRNAs are highly tolerant to exonucleases in body fluids, which makes them feasible as special molecular markers in diseases [9]. CircRNAs are also involved in diverse physiological programmes, and the dysregulation of circRNAs is associated with a wide range of diseases, including osteoporosis [9]. For example, hsa_circ_0002060 in serum/plasma was significantly upregulated in osteoporosis patients and was associated with low bone mineral density (BMD). Furthermore, hsa_circ_0002060 showed good potential for diagnosing osteoporosis with a high sensitivity and specificity [10]. Hsa_circ_0006393 promotes osteogenesis by sponging miR-145-5p and upregulating FOXO1 in osteoporosis [11]. Hsa_circ_0016624 prevents osteoporosis and promotes BMP2 expression by sponging miR-98 [12]. Hsa_circ_0076906 promotes osteogenesis and alleviates osteoporosis by acting as a sponge for miR-1305 and upregulating osteoglycin [13]. With the development of different screening methods for circRNAs, such as circRNA microarrays, circRNA sequencing, Northern blotting, and PCR-based analyses, increasing circRNAs are being identified as involved in the progression of various diseases. However, only a few circRNAs have been investigated in osteoporosis. Therefore, it is imperative to investigate the association between circRNA expression and osteoporosis development.

In the present study, we compared circRNA expression profiles of exosomes isolated from the serum of osteoporosis patients and matched healthy controls using circRNA microarray technology. Hsa_circ_0006859, one of the most upregulated circRNAs as revealed by microarray analysis, was investigated to explore its clinical significance and potential regulatory mechanism in osteoporosis. Hsa_circ_0006859 was reported to be upregulated during chondrogenic differentiation of human mesenchymal stromal cells by interacting with five possible miRNAs [14]. Hsa_circ_0006859 was reported to be upregulated in colorectal cancer [15]. Hsa_circ_0006859 was also reported to be downregulated in bladder cancer [16]. However, these reports barely discuss the role of hsa_circ_0006859 on miRNAs and the related cell signalling in their research on human diseases. In the present study, our results indicated that exosomal circRNAs were differentially expressed between osteoporosis patients and healthy controls. Therefore, Hsa_circ_0006859 is a potential biomarker for postmenopausal osteoporosis. Furthermore, hsa_circ_0006859 controls the balance between osteogenesis and adipogenesis by sponging miR-431-5p to regulate ROCK1.

## Methods

### Patients and samples

This study was approved by the Ethics Committee of the Third Affiliated Hospital of Soochow University (No.CZYY2020032). Each participant signed an informed consent document. All participants were postmenopausal women and were consecutively recruited at Osteoporosis Outpatient Clinic in the Third Affiliated Hospital of Soochow University from January 2017 to June 2019. Every participant signed the informed consent. Lumbar spine L1-L4 BMD was evaluated by dual energy X-ray absorptiometry (DXA) (Hologic Discovery Wi, Hologic, USA) according to standard operation instructions. *T*-score is the standard deviation of the BMD value from the mean. Recruited participants were classified as the osteoporosis group, defined by lumbar spine (L1-L4) BMD *T*-score ≤ − 2.5; the osteopenia group, defined by lumbar spine (L1-L4) BMD T-score ≤ − 1.0 and > − 2.5; and the control group, defined by lumbar spine (L1-L4) BMD T-score ≥ − 1.0. All recruited participants were newly diagnosed with osteoporosis or osteopenia. Participants with other relevant chronic diseases were excluded. The detailed characteristics of study subjects are summarized in Table 1. Thirty of the 58 participants in the osteoporosis group received anti-osteoporotic treatment using alendronate supplemented with calcium and vitamin D for 9 months. Peripheral blood was collected from each participant at initial diagnosis and from each participant who received anti-osteoporotic treatment at 9 months. Serum was centrifuged and collected after the blood was left for 1 h at room temperature to allow clotting. Aliquots were stored at − 80 °C until use.

**Table 1:** Demographic characteristics of the participants

	Control	Osteopenia	Osteoporosis	*P* value
Age (years)	58.32 ± 3.14	59.28 ± 4.06	60.67 ± 6.24	0.058
Body mass index (kg/m^2^)	23.8 ± 1.2	24.0 ± 2.1	24.1 ± 1.6	0.124
BMD (g/cm^2^)	0.93 ± 0.08	0.82 ± 0.11	0.65 ± 0.06	0.012
Lumbar spine (L1-L4) *T*-score	− 0.25 ± 0.04	− 1.82 ± 0.09	− 2.86 ± 0.17	< 0.0001
Lumbar spine (L1-L4) *Z*-score	− 0.26 ± 0.12	− 1.54 ± 0.03	− 2.76 ± 0.10	< 0.0001
PTH (pg/ml)	38.6 ± 12.4	36.9 ± 11.3	36.8 ± 12.2	> 0.05
25(OH) D (ng/ml)	23.6 ± 4.9	24.2 ± 3.8	23.9 ± 5.8	> 0.05
β-CTx (pg/ml)	558.6 ± 43.1	698.7 ± 52.1	808.4 ± 37.6	< 0.0001
tP1NP (ng/ml)	29.33 ± 3.21	45.22 ± 2.14	59.12 ± 4.02	0.018
Osteocalcin (ng/ml)	7.99 ± 1.08	13.55 ± 2.61	19.02 ± 3.10	0.002
Calcium (mmol/L)	2.43 ± 0.12	2.38 ± 0.07	2.39 ± 0.09	> 0.05
Inorganic phosphorus (mmol/L)	1.21 ± 0.08	1.20 ± 0.06	1.22 ± 0.04	> 0.05

### Biochemical assays

Assays for parathyroid hormone (PTH), 25-hydroxyvitamin D (25(OH)D), β-isomerized C-terminal telopeptides (β-CTx), total N-terminal procollagen of type l collagen (tP1NP), osteocalcin, calcium, and inorganic phosphorus in serum were measured by the Department of Laboratory Medicine in the hospital. PTH was analysed on UniCel DxI 800 (Beckman Coulter, USA). 25(OH) D, β-CTx, tP1NP, and osteocalcin were analysed on Cobas E601 (Roche Diagnostics, Mannheim, Germany). Calcium and inorganic phosphorus were analysed on AU5831 (Beckman Coulter, USA).

### Exosome isolation

Exosomes were isolated from serum using the miRCURY Exosome Serum/Plasma Kit (Thermo Fisher, USA) according to the manufacturer’s instructions. Then, 100 ng synthetic cel-miR-39-3p (Thermo Fisher, USA) was added as a spike-in control similar to previous reports [17, 18]. Briefly, 500 μl serum was incubated with the precipitation buffer for 1 h at 4 °C. The mixture was fully vortexed and then centrifuged at 1500*g* for 30 min at room temperature, and the supernatant was discarded to obtain the exosome precipitate. Purified exosomes were resuspended by adding resuspension buffer.

### CircRNA microarray

The circRNA microarray assay was performed on Human CircRNA Array V2 (CapitalBio Technology, Beijing, China) by CapitalBio (CapitalBio Technology, Beijing, China) as previously reported [19]. Briefly, total RNA was extracted from the exosomes, which were isolated from the serum of 3 osteoporosis patients and 3 matched controls. Extracted RNAs were digested, amplified, labelled with Cy3-dCTP, and hybridized to the microarray, according to the manufacturer’s instructions. Data were summarized, normalized, and analysed using the GeneSpring software V13.0 (Agilent, USA). The data were deposited in NCBI’s Gene Expression Omnibus under the accession number (GSE161361).

### Cell culture

Human bone marrow mesenchymal stem cells (hBMSCs) (HUXMA-01001) were obtained from Cyagen Biosciences (Soochow, China). hBMSCs were cultured in Alpha Modified Eagle’s Medium (α-MEM) (Thermo Fisher, USA) containing 10% FBS (Thermo Fisher, USA), 100 U/mL penicillin (Thermo Fisher, USA), and 100 mg/mL streptomycin (Thermo Fisher, USA) in the incubator with 5% CO_2_ and 95% humidity at 37 °C. Cells were trypsinized and passaged at 85% confluence using 0.25% trypsin-EDTA (Thermo Fisher, USA). To induce osteogenic differentiation, cells were seeded into six-well plates until they reached 70% confluence and were then cultured in osteogenic induction medium (OIM) (HUXMA-90021, Cyagen Biosciences, China) for 3 days. The medium was completely refreshed every 3 days. Cells were washed and collected after induction for 2–3 weeks. To induce adipogenic differentiation, cells were seeded into six-well plates until they reached 100% confluence and were then cultured in adipogenic induction medium (AIM) Solution A (HUXMA-90031, Cyagen Biosciences, China) for 3 days. The medium was completely replaced with AIM Solution B (Cyagen Biosciences, China) for 24 h. After three cycles of Solution A/Solution B, cells were incubated in Solution B for an additional 5 days.

### Cell transfection

Small interfering RNA (siRNA) against hsa_circ_0006859 (si-circ), siRNA against ROCK1 (si-ROCK1), siRNA negative control siRNA (si-NC), miR-431-5p mimics (miR-431 M), miR-431-5p inhibitor (miR-431I), miRNA negative control (miR-NC), pcDNA 3.1 vector containing hsa_circ_0006859 (OE-circ), pcDNA 3.1 vector containing ROCK1 without 3′-UTR (OE-ROCK1), and empty vector control (EV) were custom-designed and synthesized by Beierbo (Beierbo, China). Briefly, hBMSCs were seeded into a 6-well plate (1 × 10^6^ cells/well) and were cultured until they reached 85% confluence. Cells were then transfected with different treatments using Lipofectamine 2000 reagent (Thermo Fisher, USA) according to the manufacturer’s instructions.

### RNA extraction and quantitative real-time PCR (qRT-PCR)

Total RNA was extracted from exosomes or cells using TRIzol reagent (Thermofisher, USA). The purity and concentration of RNA was spectrophotometrically analysed using a Nanodrop One (Thermo Fisher, USA). To determine expression of circRNAs, RNase R was added to digest linear transcripts. cDNA was transcribed using the PrimeScript RT reagent kit (TaKaRa, Japan). To determine expression of miRNAs, cDNA was transcribed using the TaqMan MicroRNA Reverse Transcription Kit (Thermo Fisher, USA). To determine expression of mRNAs, cDNA was transcribed using the First Strand cDNA Synthesis Kit (Beyotime, China). PCR was performed on an ABI 7500 Real-Time PCR system (Thermo Fisher, USA) using the TB Green Premix Ex Taq II (Taraka, Japan). Cel-miR-39 was the reference gene for circRNA in the exosome isolates, U6 was the reference gene for miRNA in the cell, and GAPDH was the reference gene for mRNA or circRNA in the cell. The primers used in the study were as follows: hsa_circ_0006859, Forward: 5′-ACTGTCCCGTACTCGCTGAT-3′, Reverse: 5′-CGGTTCTCATGCACACTGAC-3′; miR-431-5p, Forward: 5′-ACGCGTGTCTTGCAGGCCGT-3′, Reverse: 5′-ATCCAGTGCAGGGTCCGAGG-3′; ROCK1, Forward: 5′-ACCTGTAACCCAAGGAGATGTG-3′, Reverse: 5′-CACAATTGGCAGGAAAGTGG-3′; GAPDH, Forward: 5′-TGACCACAGTCCATGCCATCAC-3′; Reverse: 5′-GCCTGCTTCACCACCTTCTTGA-3′. U6, Forward: 5′-CTCGCTTCGGCAGCACATATACTA-3′; Reverse: 5′-ACGAATTTGCGTGTCATCCTTGC-3′. Each experiment was repeated independently at least three times.

### Alkaline phosphatase (ALP) activity assay

ALP activity was detected using the Alkaline Phosphatase Assay Kit (Jiancheng, China) according to standard operation protocol. Absorbance was determined at 520 nm by BioTek Synergy 2 (BioTek, USA). Protein concentrations were analysed using the BCA Protein Assay Kit (Beyotime, China). The final value was calculated after normalization to protein content. Each experiment was repeated independently at least three times.

### Alizarin red staining (ARS) assay

Cells were fixed in standard solution (PBS+4% paraformaldehyde) at room temperature and were stained with 0.1% Alizarin red (pH = 4.2, Solarbio, China) according to the manufacturer’s procedure. Cells were washed with PBS three times to remove unbound dye. The cells were observed and imaged under the IX71 microscope (Olympus, Japan). To quantify mineralization, the bound dye was dissolved in the eluent containing 10% cetylpyridinium chloride and then transferred to a 96-well plate. Absorbance was measured at 570 nm using a BioTek Synergy 2 (BioTek, USA). Each experiment was independently repeated at least three times.

### Oil Red O staining

Cells were washed twice with PBS and then fixed in standard solution for 30 min at room temperature. Cells were then incubated with Oil Red O solution (Jiancheng, China) for 1 h at room temperature. Then, stained cells were washed with PBS three times and visualized under an IX71 microscope (Olympus, Japan).

### Bioinformatics analysis and dual-luciferase assay

The bioinformatics database Circular RNA Interactome was used to search for potential miRNAs that may be targeted by hsa_circ_0006859 [20]. Seventy-one miRNAs were predicted to be the targets of hsa_circ_0006859. As miR-431-5p was found to be correlated with adipogenic differentiation of hBMSCs [21], miR-431-5p was selected as one of the possible targets of hsa_circ_0006859. The two predicted binding sites on miR-431-5p targeted by hsa_circ_0006859 were at position 1668–1674 (#1) and position 401–407 (#2). Target genes of miR-431-5p were predicted by two independent databases, TargetScan [22] and DIANA Tools [23]. Though IRS2 was proven to be one of the direct target genes of miR-431-5p [21], genes directly targeted by miR-431-5p were screened since one miRNA may target many genes. Target genes predicted by both computational algorithms were selected, and a literature review was performed to further select genes associated with osteoporosis. ROCK1 was selected as one of the miR-431-5p target genes. The prediction was further verified in vitro by luciferase assay. Two hsa_circ_0006859 fragments and the ROCK1 3′-UTR possessing wild type (Wt) or mutant (Mut) seed region were synthesized and cloned into psiCHECK-2 vectors (Promega, USA), termed circ-#1-Wt, circ-#1-Mut, circ-#2-Wt, circ-#2-Mut, ROCK1-Wt, and ROCK1-Mut. After hBMSCs reached 85% confluence, these constructed reporter plasmids were co-transfected with miR-431-5p mimics using Lipofectamine 2000 reagent (Thermo Fisher, USA) according to the manufacturer’s protocol. After transfection for 24 h, the fluorescence in each well was detected using the Dual Luciferase Reporter Assay System (Thermo Fisher, USA).

### RNA pull-down assay

Biotinylated miR-NC, miR-431-5p-Wt, and miR-431-5p-Mut were transfected into hBMSCs with Lipofectamine 2000 reagent (Thermo Fisher, USA). After sonication, cell lysates were collected and incubated with Dynabeads M-280 Streptavidin (Thermo Fisher, USA) according to standard operation. Bound RNAs were purified using TRIzol reagent (Thermo Fisher, USA), and hsa_circ_0006859 was detected by qRT-PCR.

### Western blot

Total protein was extracted from hBMSCs after different treatments using RIPA lysis buffer (Beyotime, China) supplemented with protease inhibitors (Thermo Fisher, USA), and protein concentration was quantified using the BCA Protein Assay Kit (Beyotime, China). SDS-PAGE electrophoresis was performed to separate proteins. The gel was then transferred onto a PVDF membrane (Thermo Fisher, USA). The membrane was blocked in 5% non-fat milk for 1 h at room temperature and was subsequently incubated with specific primary antibody at 4 °C overnight. The membrane was rinsed three times with TBST and then incubated with specific secondary antibody for 1 h at room temperature. The membrane was washed, incubated with an enhanced chemiluminescence reagent (Thermo Fisher, USA), and observed on the ChemiDoc Touch Imaging System (Bio-Rad, USA). All experiments were independently repeated three times.

### Statistical analysis

Each experiment was repeated three times. All data are expressed as the mean ± SEM. Statistical analysis was performed using GraphPad Prism 7.0. Student’s *t* test was used to test the difference between two groups. The one-way analysis of variance (ANOVA) was used to test the difference between three or more groups. Receiver operating characteristic (ROC) analysis was performed to evaluate the diagnostic ability of gene expression for disease prediction. Differences were considered statistically significant when *P* < 0.05.

## Results

### CircRNAs in exosome are differentially expressed in osteoporosis patients

**Fig. 1:**
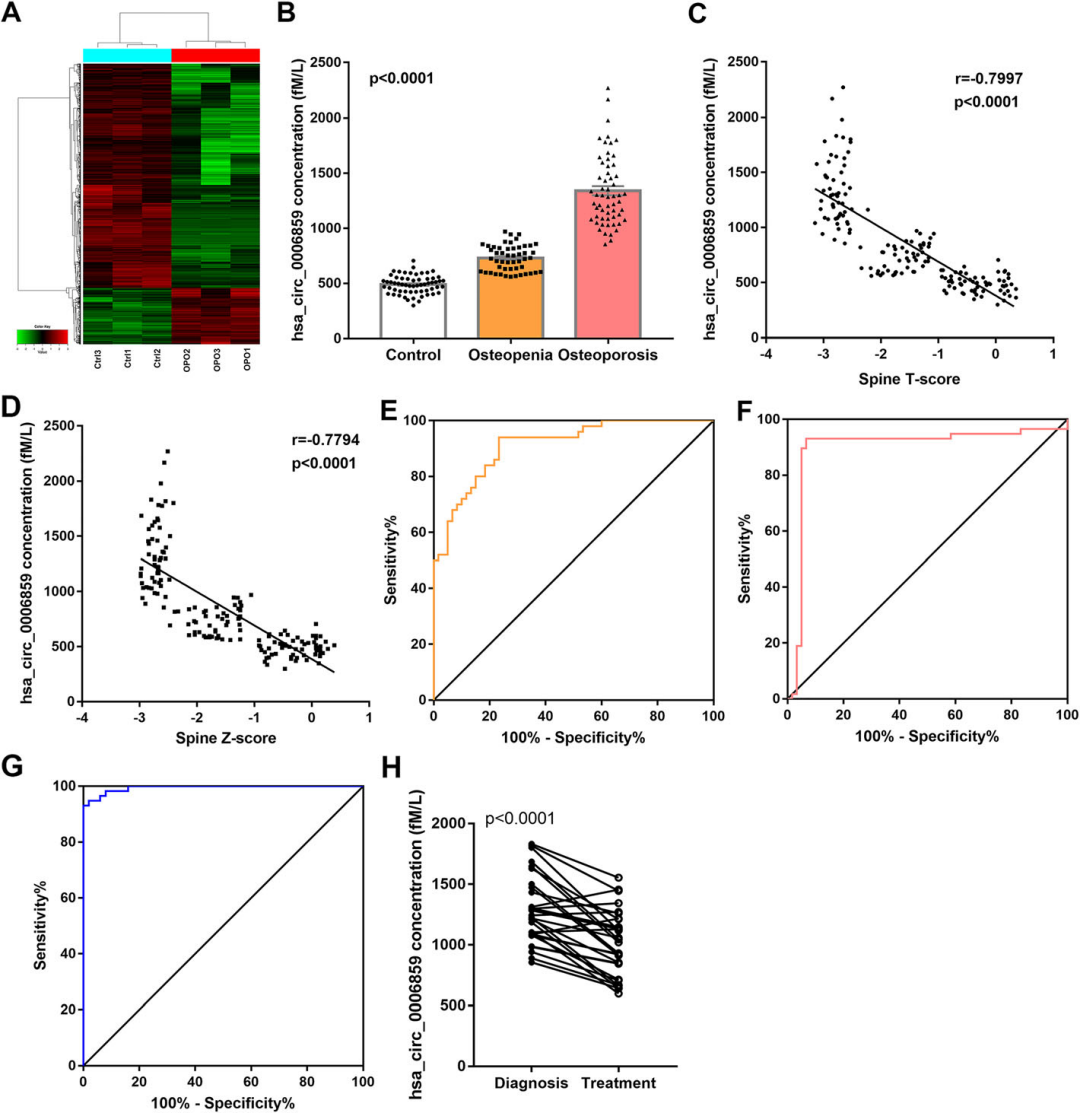
hsa_circ_0006859 in exosomes is upregulated in osteoporosis patients. **a** Heat map of circRNA microarray profile in osteoporosis patients (*n* = 3) and healthy controls (*n* = 3). **b** Exosomal hsa_circ_0006859 expression in patients with osteopenia or osteoporosis and in healthy controls. **c** The correlation between hsa_circ_0006859 expression and spine *T*-score. **d** The correlation between hsa_circ_0006859 expression and spine *Z*-score. **e** ROC curve analysis of exosomal hsa_circ_0006859 to differentiate osteopenia patients from healthy controls. **f** ROC curve analysis of exosomal hsa_circ_0006859 to differentiate osteoporosis patients from healthy controls. **g** ROC curve analysis of exosomal hsa_circ_0006859 to differentiate osteoporosis patients from osteopenia patients. **h** Expression levels of exosomal hsa_circ_0006859 between samples from OPO patients at diagnosis and samples from patients who received anti-osteoporotic treatment for 9 months

The circRNA expression profiles of exosomes isolated from the serum of osteoporosis patients (*N* = 3) and matched healthy controls (*N* = 3) were examined by circRNA microarray. To select potential differentially expressed circRNAs, the selection criteria were as follows: (1) circRNA was detected among all samples, (2) fold change > 4 or < 0.25, and (3) *P* < 0.05 according to the *t* test. The results showed that circRNAs in exosomes were differentially expressed between the two groups (Fig. 1a). Five hundred eighty-nine circRNAs were differentially expressed between the two groups, of which 376 circRNAs were upregulated and 213 were downregulated in osteoporosis patients. The five most upregulated or downregulated circRNAs in osteoporosis patients are listed in Supplementary Table 1. Though hsa_circ_006859 was the second most upregulated circRNA, hsa_circ_006859 was selected for further study due to hsa_circ_0006859 being found to exist in human serum [24], cells [25], and tissues [26], and it was upregulated in colorectal cancer tissues compared to the adjacent non-tumour tissues [26], while there are no reports concerning the role of hsa_circ_0047341. Thus, we chose to examine hsa_circ_006859. In total, these results indicated that circRNAs in exosome are differentially expressed in osteoporosis patients, and hsa_circ_006859 was selected for further experiments.

### hsa_circ_0006859 in exosomes is upregulated in osteoporosis patients

The demographic characteristics of participants included in this study are shown in Table 1. A total of 168 postmenopausal women from the Third Affiliated Hospital of Soochow University were recruited, including 60 participants with normal bone mass as healthy controls, 50 participants with osteopenia, and 58 participants with osteoporosis. No significant difference was observed in age, body mass index (BMI), PTH, 25(OH) D, calcium, or inorganic phosphorus among the three groups. BMD, *T*-score, *Z*-score, β-CTx, tP1NP, and osteocalcin were significantly different among the osteoporosis, osteopenia, and healthy groups, indicating that participants with osteopenia or osteoporosis exhibited worsening bone parameters compared to the control group, especially the osteoporosis group (Table 1). The 3 osteoporosis patients and 3 healthy controls for circRNA microarray were not included. To determine whether hsa_circ_0006859 is involved in the development of osteoporosis, expression levels of hsa_circ_0006859 in exosomes were detected using qRT-PCR. Expression levels of hsa_circ_0006859 in exosomes were significantly higher in osteoporosis and osteopenia patients compared to non-osteoporotic controls (*p* < 0.0001, Fig. 1b). Expression levels of hsa_circ_0006859 were also significantly higher in osteoporosis patients than in osteopenia patients (*p* < 0.0001, Fig. 1b). We next analysed the relationship between expression levels of hsa_circ_0006859 in exosome and clinical parameters. Expression levels of hsa_circ_0006859 in exosome showed a strong negative correlation with L1-L4 spine *T*-score (Fig. 1c) and L1-L4 spine *Z*-score (Fig. 1d) across the entire cohort. To assess the diagnostic value of exosomal hsa_circ_0006859 to discriminate osteoporosis or osteopenia patients from healthy controls, ROC curve analysis was conducted. Hsa_circ_0006859 differentiated osteopenia patients from healthy controls with an AUC of 0.913 (95% CI 0.8617–0.9643, *p* < 0.0001), 94.00% sensitivity, and 76.67% specificity (Fig. 1e). Hsa_circ_0006859 also significantly differentiated osteoporosis patients from healthy controls with an AUC = 0.8974 (95% CI 0.8248–0.97, *p* < 0.0001), 93.1% sensitivity, and 93.33% specificity (Fig. 1f). Furthermore, hsa_circ_0006859 significantly differentiated osteoporosis patients from osteopenia patients with an AUC = 0.8873 (95% CI 0.8209–0.9536, *p* < 0.0001), 75.0% sensitivity, and 93.3% specificity (Fig. 1g). Expression levels of hsa_circ_0006859 in exosomes extracted from the serum of OPO patients after anti-osteoporotic treatment for 9 months were also significantly downregulated compared to those at diagnosis (Fig. 1h). These results suggested that hsa_circ_0006859 in exosomes was upregulated in patients with osteopenia or osteoporosis, and it may distinguish these patients from healthy controls, suggesting its potential for the diagnosis of osteoporosis and its critical role in the pathophysiology of osteoporosis.

### hsa_circ_0006859 suppresses osteoblastic differentiation

**Fig. 2:**
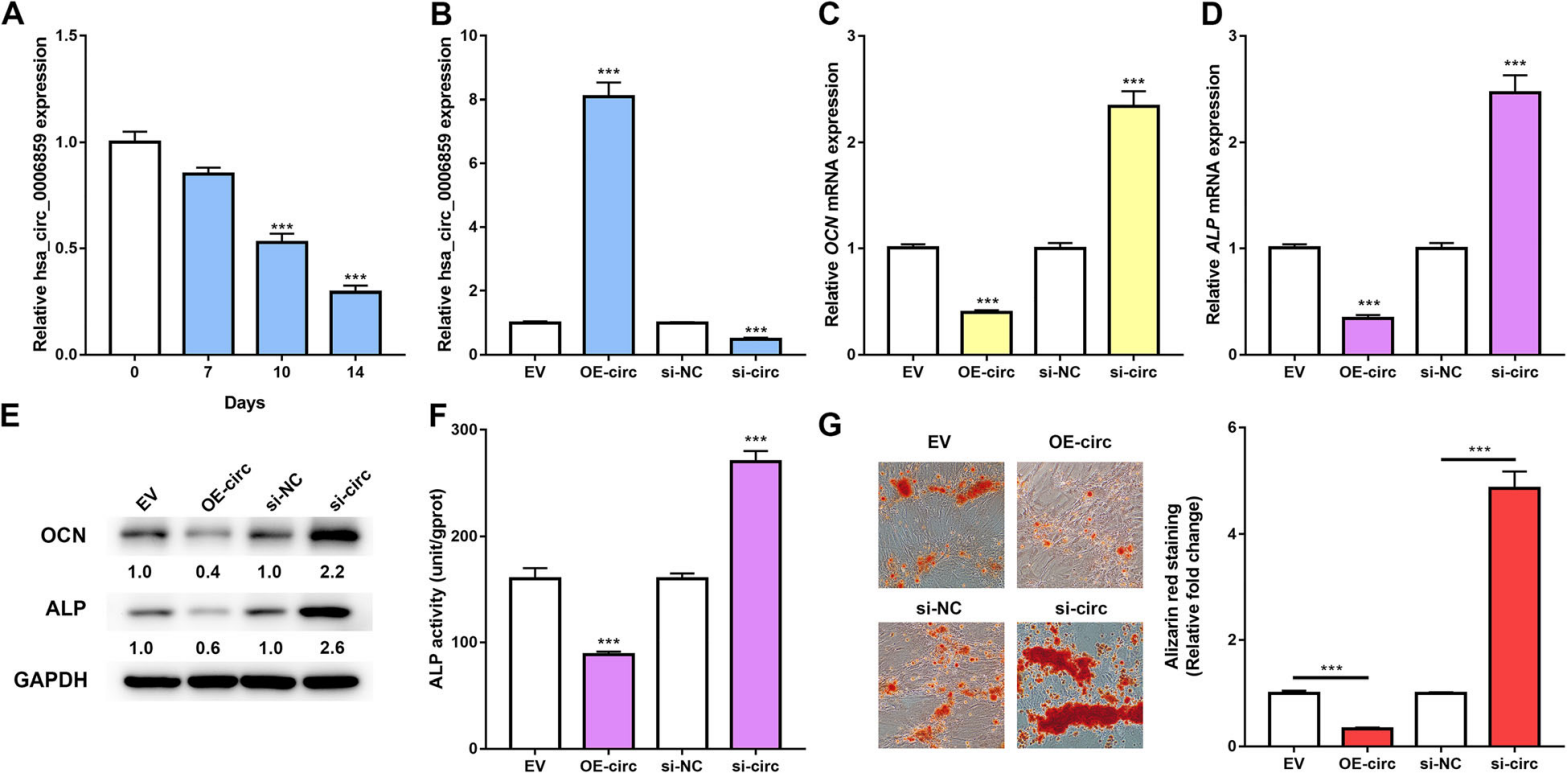
hsa_circ_0006859 suppresses osteoblastic differentiation. **a** Relative hsa_circ_0006859 expression in hBMSCs during osteogenic differentiation. **b** Relative hsa_circ_0006859 expression in hBMSCs after transfection with OE-circ or si-circ. **c** Relative *OCN* mRNA expression in hBMSC after transfection with OE-circ or si-circ. **d** Relative *ALP* mRNA expression in hBMSCs after transfection with OE-circ or si-circ. **e** Western blot analysis of OCN and ALP expression in hBMSCs after transfection with OE-circ or si-circ. **f** ALP activity in hBMSCs after transfection with OE-circ or si-circ. **g** Alizarin red staining was used to detect the mineralization ability of hBMSCs after transfection with OE-circ or si-circ. ***P < 0.0001

To investigate the function of hsa_circ_0006859 in osteoporosis, expression of hsa_circ_0006859 during osteoblastic differentiation of hBMSCs induced by OIM was examined by qRT-PCR at indicated days (0, 7, 10, and 14 days). Hsa_circ_0006859 in cell was gradually decreased during osteoblastic differentiation in hBMSCs (Fig. 2a). The OE-circ or si-circ was transfected into hBMSCs to overexpress or silence hsa_circ_0006859, respectively. Expression of hsa_circ_0006859 was significantly increased by approximately 8-fold after OE-circ transfection and was decreased about half-fold after si-circ transfection, indicating that the cell transfection was successful (Fig. 2b). Osteocalcin (OCN) and alkaline phosphatase (ALP) are commonly used osteoblast differentiation markers. Relative mRNA expression levels of *OCN* (Fig. 2c) and *ALP* (Fig. 2d) were significantly decreased in hBMSCs transfected with OE-circ, while relative mRNA expression levels of *OCN* (Fig. 2c) and *ALP* (Fig. 2d) were significantly increased in hBMSCs transfected with si-circ after induction of osteoblastic differentiation for 10 days. Hsa_circ_0006859 overexpression significantly decreased protein levels of OCN and ALP in hBMSCs, while hsa_circ_0006859 inhibition had the opposite effect, which was illustrated by western blot analysis after hBMSCs were cultured in OIM for 10 days (Fig. 2e). In addition, ALP activity and ARS staining assays were performed to evaluate the effects of hsa_circ_0006859 on the osteogenic process. ALP activity in hBMSCs transfected with OE-circ was significantly decreased, while ALP activity was significantly enhanced in hBMSCs transfected with si-circ (Fig. 2f). Calcified nodules were markedly decreased following hsa_circ_0006859 overexpression, while calcified nodules were significantly increased following hsa_circ_0006859 suppression (Fig. 2g). These results imply that hsa_circ_0006859 suppresses osteoblastic differentiation of hBMSCs.

### Hsa_circ_0006859 promotes adipogenic differentiation

**Fig. 3:**
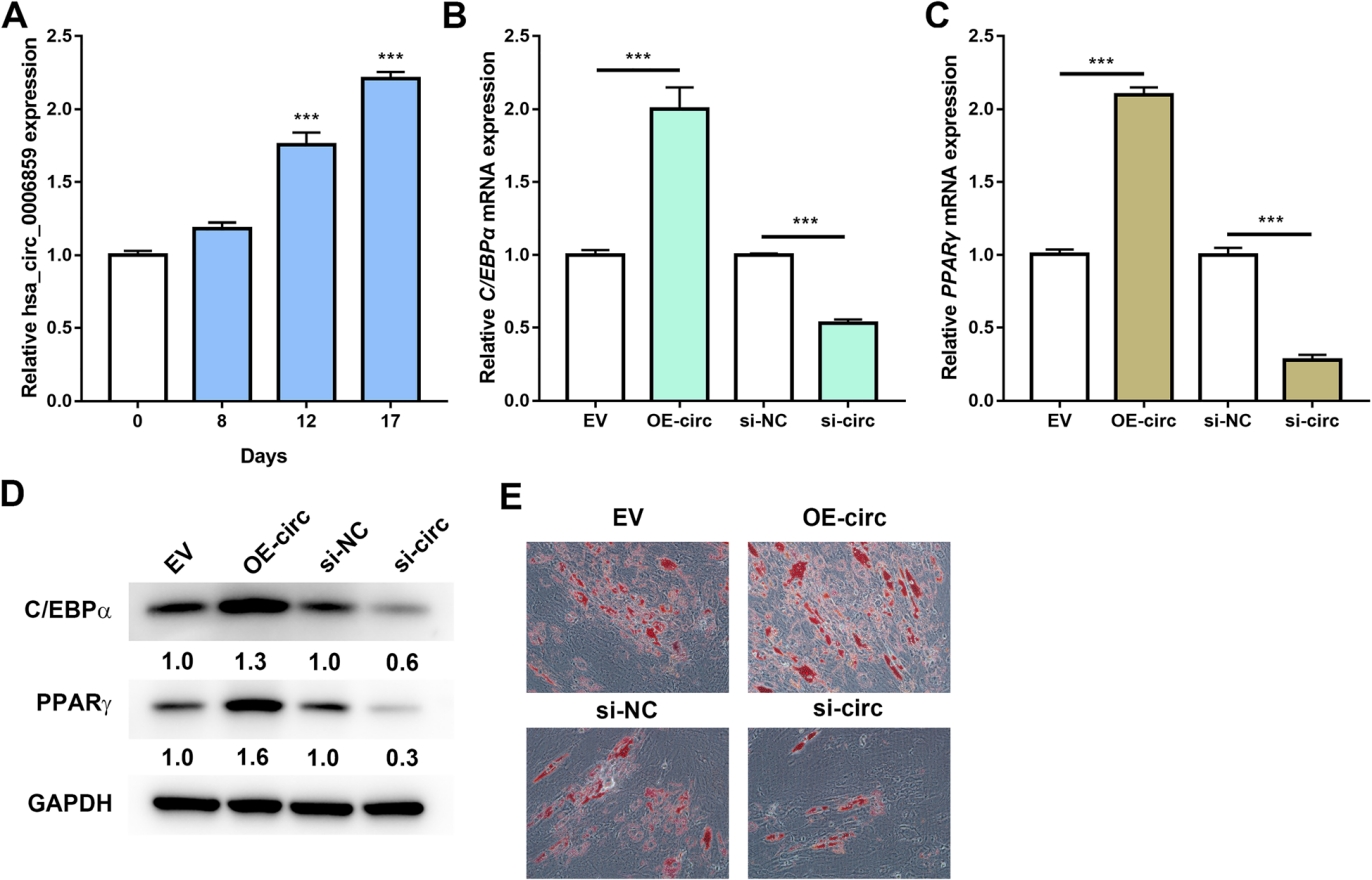
hsa_circ_0006859 promotes adipogenic differentiation. **a** Relative hsa_circ_0006859 expression in hBMSCs during adipogenic differentiation. **b** Relative *C/EBPα* mRNA expression in hBMSC after transfection with OE-circ or si-circ. **c** Relative *PPARγ* mRNA expression in hBMSC after transfection with OE-circ or si-circ. **d** Western blot analysis of C/EBPα and PPARγ expression in hBMSCs after transfection with OE-circ or si-circ. **e** Oil Red O staining of hBMSCs after transfection with OE-circ or si-circ

We next focused on the biological function of hsa_circ_0006859 in adipogenic differentiation. As shown in Fig. 3a, hsa_circ_0006859 gradually increased during adipogenic differentiation in hBMSCs. The mRNA expression levels of adipogenic markers C/EBPα and PPARγ were examined by qRT-PCR after adipogenic differentiation for 12 days. hBMSCs transfected with OE-circ exhibited higher mRNA expression levels of *C/EBPα* (Fig. 3b) and *PPARγ* (Fig. 3c) compared to EV, while hBMSCs transfected with si-circ presented lower mRNA expression levels of *C/EBPα* (Fig. 3b) and *PPARγ* (Fig. 3c) compared to si-NC. Protein levels of C/EBPα and PPARγ were examined by western blot analysis, and their changes were similar to the mRNA changes (Fig. 3d). Furthermore, overexpression of hsa_circ_0006859 by OE-circ enhanced the formation of lipid droplets, while suppression of hsa_circ_0006859 by si-circ attenuated adipogenic differentiation of hBMSCs as indicated by Oil red O staining (Fig. 3e). Therefore, these results indicate that hsa_circ_0006859 enhances adipogenic differentiation of hBMSCs.

### hsa_circ_0006859 directly binds to miR-431-5p

**Fig. 4:**
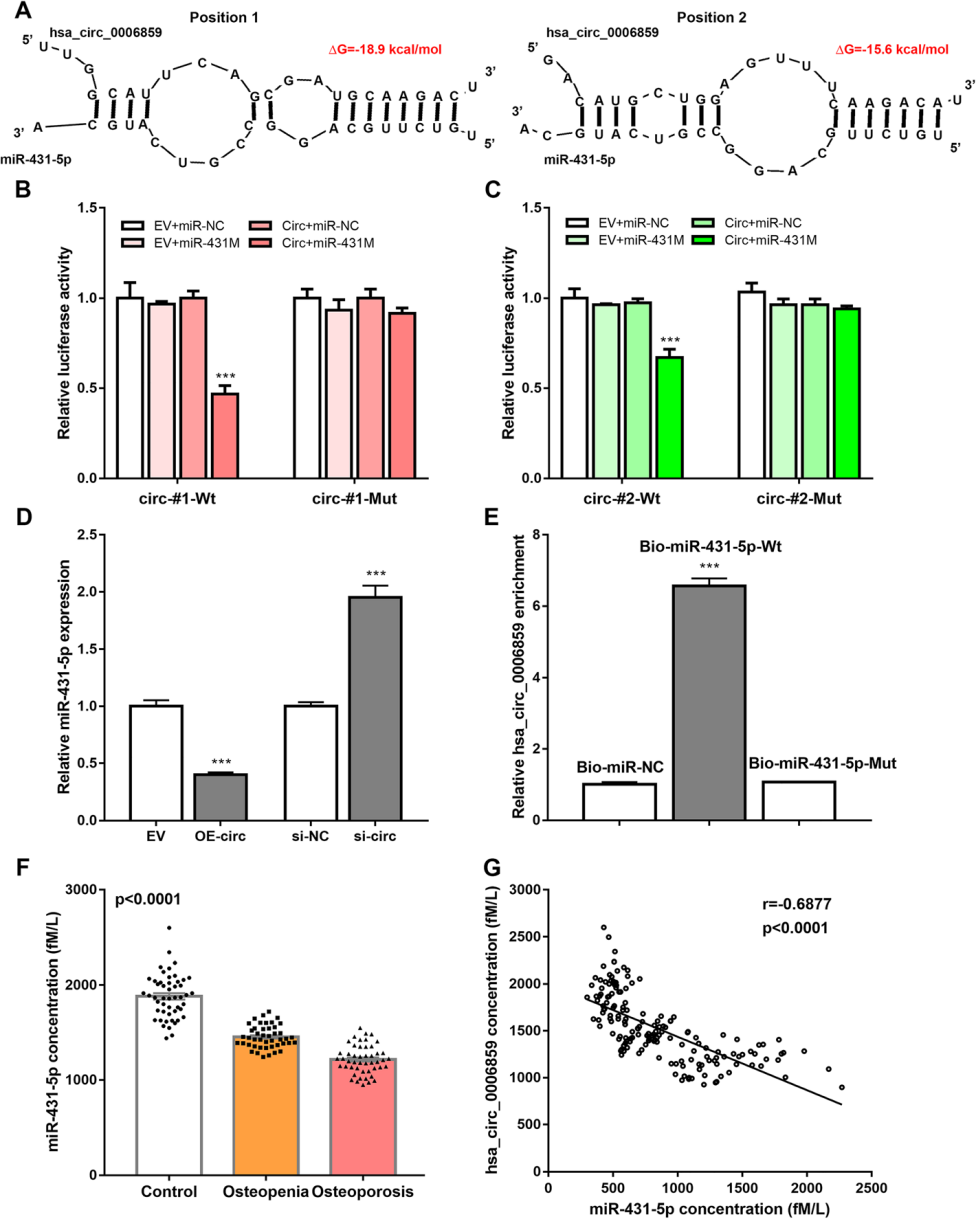
hsa_circ_0006859 directly binds to miR-431-5p. **a** Bioinformatics analysis of the predicted binding sites between hsa_circ_0006859 and miR-431-5p. **b** Luciferase activity of hsa_circ_0006859 circ-#1-Wt and circ-#1-Mut upon miR-431-5p overexpression. **c** Luciferase activity of hsa_circ_0006859 circ-#2-Wt and circ-#2-Mut upon miR-431-5p overexpression. **d** Relative miR-431-5p expression in hBMSC after transfection with OE-circ or si-circ. **e** RNA pull-down assay of the interaction between hsa_circ_0006859 and miR-431-5p

Given that circRNAs act as miRNA sponges to negatively regulate miRNA activity [27], the regulatory function of hsa_circ_0006859 at the post-transcriptional level was explored. Based on the bioinformatics analysis and literature review, miR-431-5p may directly bind to hsa_circ_0006859. Two potential miR-431-5p binding sites were identified in the hsa_circ_0006859 sequence at position 1668–1674 and position 401–407 (Fig. 4a). The minimum free energy values of the two hybrids were − 18.9 kcal/mol and − 15.6 kcal/mol (Fig. 4a). Direct interactions between hsa_circ_0006859 and miR-431-5p were verified by dual luciferase reporter assays. circ-#1-Wt, circ-#1-Mut, circ-#2-Wt, or circ-#2-Mut was co-transfected with miR-431 M into hBMSCs. The luciferase activity of circ-#1-Wt (Fig. 4b) or circ-#2-Wt (Fig. 4c) was significantly inhibited by miR-431 M, while the luciferase activity of circ-#1-Mut (Fig. 4b) or circ-#2-Mut (Fig. 4c) was not significantly changed by miR-431 M (Fig. 4c). Moreover, expression levels of miR-431-5p in hBMSCs transfected with hsa_circ_0006859 overexpression vector or hsa_circ_0006859 siRNA were determined by qRT-PCR. The expression of miR-431-5p was significantly downregulated by OE-circ, while it was significantly upregulated by si-circ (Fig. 4d). The RNA pull-down assay was performed to further verify the interaction between hsa_circ_0006859 and miR-431-5p. Relative hsa_circ_0006859 enrichment was significantly higher in the Bio-miR-431-5p-Wt group than in the Bio-miR-431-5p-Mut group or the Bio-miR-NC group (Fig. 4e). Next, expression of miR-431-5p in exosomes was detected using qRT-PCR. Expression of miR-431-5p in exosomes was significantly lower in osteoporosis patients compared to osteopenia patients and non-osteoporotic controls (Fig. 4f). The correlation between hsa_circ_0006859 expression and miR-431-5p expression in the exosomes of clinical samples was further illustrated using Pearson’s correlation analysis. There was an inverse correlation between hsa_circ_0006859 expression and miR-431-5p expression in exosomes (Fig. 4g). These findings support the conclusion that hsa_circ_0006859 directly sponges miR-431-5p.

### hsa_circ_0006859 acts as a competing endogenous RNA (ceRNA) to promote ROCK1 expression

**Fig. 5:**
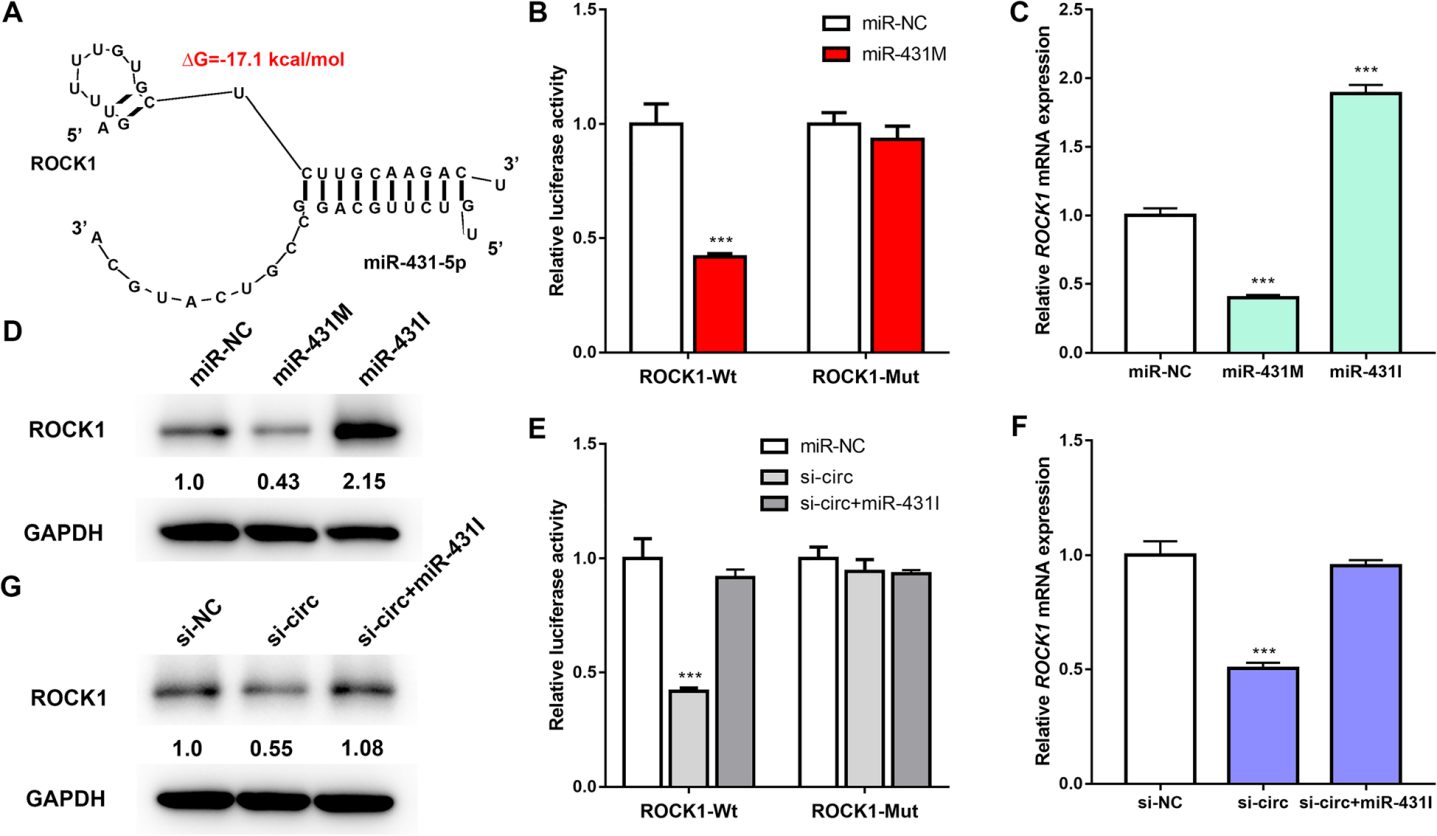
hsa_circ_0006859 acts as a competing endogenous RNA (ceRNA) to promote ROCK1 expression. **a** Bioinformatics analysis of the predicted binding sites between miR-431-5p and ROCK1. **b** Luciferase activity of ROCK1-Wt and ROCK1-Mut upon miR-431-5p overexpression. **c** Relative *ROCK1* mRNA expression in hBMSCs after transfection with miR-431 M or miR-431I. **d** Western blot analysis of ROCK1 expression in hBMSCs after transfection with miR-431 M or miR-431I. **e** Luciferase activity of ROCK1-Wt and ROCK1-Mut in hBMSCs after transfection with miR-NC, si-circ or si-circ+miR-431I. **f** Relative *ROCK1* mRNA expression in hBMSCs after transfection with miR-NC, si-circ, or si-circ+miR-431I

In this experiment, two biological algorithms, TargetScan and DIANA, were used to predict potential target genes that may share the binding sites of miR-431-5p matched in hsa_circ_0006859. ROCK1 was identified as a potential target of miR-431-5p (Fig. 5a). To identify whether miR-431-5p directly targets ROCK1 3′-UTR, Wt-ROCK1 or Mut-ROCK1 was co-transfected with miR-431-5p mimics into hBMSCs. The dual-luciferase assay showed that the luciferase activity of ROCK1-Wt was significantly inhibited by miR-431 M, while ROCK1-Mut relieved the effect induced by miR-431 M (Fig. 5b). Moreover, mRNA expression of *ROCK1* and protein expression of ROCK1 in hBMSCs transfected with miR-431 M or miR-431I were determined by qRT-PCR and western blot analysis. The qRT-PCR results demonstrated that mRNA expression of ROCK1 was significantly upregulated by miR-431I and significantly downregulated by miR-431 M (Fig. 5c). Western blot results showed that protein expression of ROCK1 was significantly downregulated by miR-431 M and significantly upregulated by miR-431I (Fig. 5d). These findings support the conclusion that ROCK1 is a direct target gene of miR-431-5p. Moreover, the role of hsa_circ_0006859 in ROCK1 regulation was investigated. The dual-luciferase assay demonstrated that the luciferase activity of ROCK1-Wt was decreased by hsa_circ_0006859 siRNA, while the reduced luciferase activity was rescued by miR-431I (Fig. 5e). However, the luciferase activity of ROCK1-Mut was barely changed by si-circ or si-circ+miR-431I (Fig. 5e). mRNA expression levels of ROCK1 (Fig. 5f) and protein expression levels of ROCK1 (Fig. 5g) were significantly decreased by hsa_circ_0006859 knockdown and were rescued by miR-431I. Taken together, these results suggest that hsa_circ_0006859 promotes ROCK1 expression by competitively binding to miR-431-5p in hBMSCs.

### hsa_circ_0006859 suppresses osteogenesis and promotes adipogenesis by sponging miR-431-5p to upregulate ROCK1

**Fig. 6:**
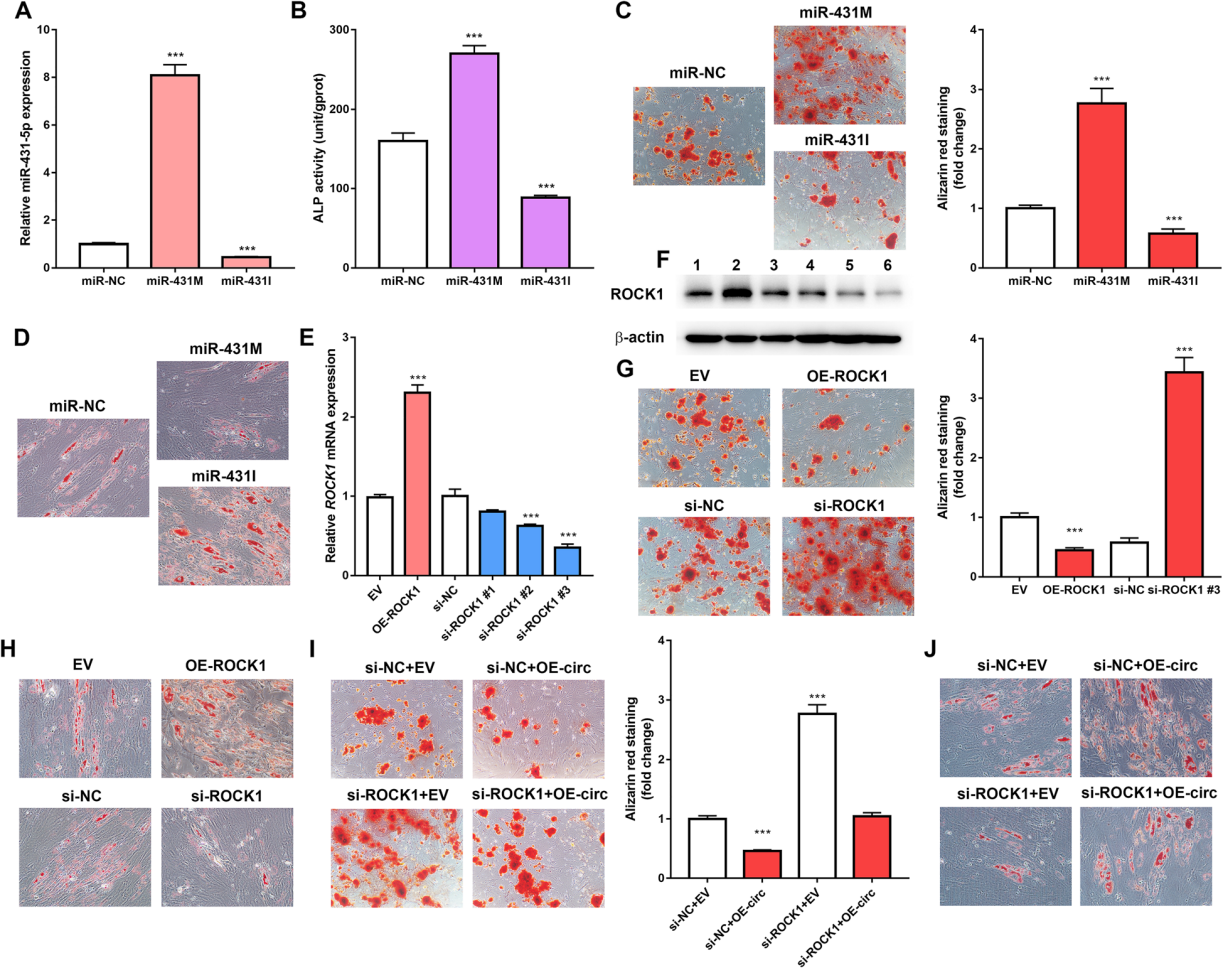
hsa_circ_0006859 suppresses osteogenesis and promotes adipogenesis by sponging miR-431-5p to upregulate ROCK1. **a** Relative miR-431-5p expression in hBMSCs after transfection with miR-431 M or miR-431I. **b** ALP activity in hBMSCs after transfection with miR-431 M or miR-431I. **c** Alizarin red staining was used to detect the mineralization ability of hBMSCs after transfection with miR-431 M or miR-431I. **d** Oil Red O staining of hBMSCs after transfection with miR-431 M or miR-431I. **e** Relative *ROCK1* mRNA expression in hBMSCs after transfection with OE-ROCK1, si-ROCK1 #1, si-ROCK1 #2, or si-ROCK1 #3. **f** Western blot analysis of ROCK1 expression in hBMSCs after transfection with OE-ROCK1, si-ROCK1 #1, si-ROCK1 #2, or si-ROCK1 #3. **g** Alizarin red staining of hBMSCs after transfection with OE-ROCK1 or si-ROCK1. **h** Oil Red O staining of hBMSCs after transfection with OE-ROCK1 or si-ROCK1. **i** Alizarin red staining of hBMSCs after transfection with si-NC+EV, si-NC+OE-circ, si-ROCK1+EV, or si-ROCK1+OE-circ. **j** Oil Red O staining of hBMSCs after transfection with si-NC+EV, si-NC+OE-circ, si-ROCK1+EV, or si-ROCK1+OE-circ

To investigate the function of miR-431-5p in osteoporosis, miR-431 M or miR-431I was transfected into hBMSCs to overexpress or silence miR-431-5p, respectively. Expression of miR-431-5p was significantly increased by approximately 8-fold after miR-431 M transfection and by about half-fold decreased after miR-431I transfection, indicating that the cell transfection was successful (Fig. 6a). ALP activity and ARS staining assays were performed to evaluate the effects of miR-431-5p on the osteogenic process. ALP activity in hBMSCs transfected with miR-431 M was significantly enhanced, while the ALP activity was significantly suppressed in hBMSCs transfected with miR-431I (Fig. 6b). Calcified nodules were increased following miR-431-5p overexpression and decreased following miR-431-5p suppression (Fig. 6c). We next focused on the biological function of miR-431-5p in adipogenic differentiation. Overexpression of miR-431-5p by miR-431 M weakened the formation of lipid droplets, while suppression of miR-431-5p by miR-431I strengthened the adipogenic differentiation of hBMSCs as indicated by the Oil red O staining (Fig. 6d). Therefore, these results indicate that miR-431-5p promotes osteoblastic differentiation and inhibits adipogenic differentiation of hBMSCs.

Next, we investigated the functional role of ROCK1 in the regulation of osteoblastic differentiation and adipogenic differentiation. OE-ROCK1 was transfected into hBMSCs to overexpress ROCK1, while si-ROCK1 was transfected into hBMSCs to inhibit ROCK1. qRT-PCR analysis showed that OE-ROCK1 significantly enhanced ROCK1 mRNA expression, while si-ROCK1 #2 and #3 significantly decreased ROCK1 mRNA expression in hBMSCs (Fig. 6e). Western blot analysis demonstrated that ROCK1 protein expression was significantly enhanced by ROCK1 OE transfection, while ROCK1 protein expression was significantly decreased by ROCK1 si-ROCK1 #2 and #3 in hBMSCs (Fig. 6f). As si-ROCK1 #3 was very effective in knocking down ROCK1, both at the mRNA and protein levels, it was selected in the following experiments. ROCK1 overexpression significantly decreased the mineralized nodule formation, while ROCK1 knockdown significantly enhanced mineralized nodule formation compared to their respective controls (Fig. 6g). Furthermore, ROCK1 overexpression significantly enhanced the formation of lipid droplets, while ROCK1 knockdown significantly decreased the formation of lipid droplets compared to their respective controls (Fig. 6h). These results indicate that ROCK1 suppresses osteoblastic differentiation and promotes adipogenic differentiation of hBMSCs.

Because hsa_circ_0006859 was found to directly bind with miR-431-5p and ROCK1 was directly targeted by miR-431-5p, we speculated that the regulatory effects of hsa_circ_0006859 on hBMSCs osteogenesis and adipogenesis were mediated by the miR-431-5p/ROCK1 axis. ARS staining revealed that the reduced osteoblastic differentiation caused by hsa_circ_0006859 overexpression was partially reversed by ROCK1 knockdown (Fig. 6i). Oil red O staining revealed that the enhanced adipogenic differentiation caused by hsa_circ_0006859 overexpression was partially reversed by ROCK1 knockdown (Fig. 6j). Collectively, hsa_circ_0006859 suppressed osteogenesis and promoted adipogenesis via sponging miR-431-5p to upregulate ROCK1.

## Discussion

Osteoporosis is a skeletal disorder characterized by impaired bone density and quality, which increase the risk of bone fractures, especially in postmenopausal women [28]. Many studies have shown that circRNAs are involved in the progression of a variety of pathological conditions and are likely to be new potential clinical biomarkers or therapeutic targets in human diseases [5, 29]. In the present study, we found that circRNAs in exosome were differentially expressed in osteoporosis patients compared to healthy controls. Hsa_circ_0006859 in exosomes was significantly upregulated in osteoporosis patients and had a good discrimination to distinguish osteoporosis patients from healthy controls. Furthermore, hsa_circ_0006859 inhibited osteoblastic differentiation and promoted adipogenic differentiation of hBMSCs by sponging miR-431-5p to upregulate ROCK1 in vitro. Taken together, our results demonstrated that hsa_circ_0006859 may be a potential biomarker for detection and disease progression of osteoporosis and may represent a promising therapeutic target for the treatment of osteoporosis in postmenopausal women.

CircRNAs are abundant in exosomes, and more than one thousand exosomal circRNAs have been identified in human serum [7]. CircRNAs are ideal diagnostic biomarkers in human diseases due to their universality (prevailing expression), conservatism (evolutionally conserved), stability (highly resistant to RNase R), and specificity (tissue- and stage-specific) [5]. For example, exosomal hsa_circ_0004771 was upregulated in the serum of colorectal cancer patients and was downregulated in the serum of postoperative colorectal cancer patients. Exosomal hsa_circ_0004771 was able to differentiate colorectal cancer patients from healthy controls with high sensitivity and specificity [30]. Several serum exosomal circRNAs, such as hsa_circ_0082333, were able to distinguish patients with colon cancer from healthy controls [6]. Exosomal hsa_circ_0130810 was decreased in gastric cancer patients, and its reduction was correlated with shorter overall survival time, indicating that exosomal hsa_circ_0130810 may serve as a novel biomarker for gastric cancer [31]. Currently, the preferred technology for diagnosing osteoporosis is the DXA (dual-energy X-ray absorptiometry) scan, which measures BMD in the spine, hip, or forearm and predicts skeletal fracture risk [32]. Although DXA is valuable, it can only reveal already established changes in bone structure, which may take a long time to become detectable and can only provide limited information on disease progression and treatment efficacy [33]. To date, several circRNAs have been proposed as potentially valuable biomarkers for osteoporosis. Hsa_circ_0001275 in peripheral blood mononuclear cells was higher in osteoporosis patients when compared to healthy controls, and its expression was negatively correlated with *T*-score. ROC curve analysis showed that hsa_circ_0001275 efficiently differentiated osteoporosis patients from healthy controls [34]. Furthermore, expression of serum hsa_circ_0002060 was upregulated in osteoporosis patients compared to control groups, and its expression was associated with low BMD in osteoporosis patients. ROC analysis indicated that hsa_circ_0002060 shows potential diagnostic value for osteoporosis [10]. In the present study, exosomal hsa_circ_0006859 was upregulated in postmenopausal women with osteoporosis. Its expression in exosomes was negatively correlated with L1-L4 spine T-score and Z-score. Furthermore, hsa_circ_0006859 significantly differentiated osteoporosis patients from healthy controls. Taken together, our results suggest the clinical potential of exosomal hsa_circ_0006859 in the diagnosis of osteoporosis. To our knowledge, this is the first study in which exosomal hsa_circ_0006859 in serum was successfully identified as a potential biomarker for postmenopausal osteoporosis.

An increasing number of studies have demonstrated that circRNAs play critical roles in multiple diseases through different mechanisms [5]. Some circRNAs interact with miRNAs, which releases miRNA-mediated mRNA inhibition in osteoporosis. Plasma circRNA_0016624 is downregulated in osteoporosis. It was found to prevent osteoporosis and promote BMP2 expression by sponging miR-98 [12]. Hsa_circ_0006393 promotes osteogenesis by sponging miR-145-5p and upregulating FOXO1 in osteoporosis [11]. Hsa_circ_0028313 knockdown suppressed osteoclast differentiation and prevented bone loss via the miR-195a/CSF1 axis [35]. Hsa_circ_0076906 promoted osteogenesis and alleviated osteoporosis by acting as a sponge for miR-1305 and upregulating osteoglycin [13]. In the present study, hsa_circ_0006859 was found to suppress osteogenesis and promote adipogenesis by sponging miR-431-5p, which released the inhibitory effect of miR-431-5p on its direct target, ROCK1. This is the first study demonstrating the important role of the hsa_circ_0006859/miR-431-5p/ROCK1 axis during osteoporosis.

Multiple RNA populations have been identified in exosomes, including mRNAs and many types of non-coding RNAs, such as circRNAs, miRNAs, and lncRNAs [7]. These miRNAs in exosomes are categorized into circulating miRNAs, which are involved in many aspects of osteoporosis. Some circulating miRNAs may be applied at the prediction stage for osteoporosis. Upregulation of serum miR-21-5p and miR-23a-3p and downregulation of serum miR-125b-5p were correlated with bone loss and muscle loss in postmenopausal women [36, 37]. Circulating miR-194-5p was upregulated in the blood of postmenopausal patients with osteoporosis and was negatively correlated with *T*-scores [38]. Some circulating miRNAs may be applied at the diagnostic stage for osteoporosis. Plasma miR-19b-3p was significantly lower in osteoporosis patients and differentiated osteoporosis patients with vertebral fracture from healthy controls with relative high accuracy [39]. Serum miR-21-5p clearly distinguished healthy controls, osteopenia and osteoporosis patients with relative high sensitivity and specificity in diagnosis [37, 40]. Serum miR-30b-5p was significantly downregulated in osteopenia or osteoporosis patients and was positively correlated with BMD [41]. Some circulating miRNAs may be applied at the treatment stage for osteoporosis. Serum miR-29b-3p and miR-324-p were reduced in patients undergoing anti-resorptive therapy [42]. Serum miR-33-3p and miR-133a-3p were decreased during teriparatide treatment [43]. In our study, miR-431-5p was also found in osteoporosis serum exosomes. Furthermore, expression levels of miR-431-5p were negatively correlated with expression levels of hsa_circ_0006859 in exosomes. Our results indicate the important role of circulating miR-431-5p in osteoporosis.

Increasing evidence has suggested that miRNAs play a pivotal role in bone homeostasis. During ageing, hBMSCs reduce the ability to differentiate into osteoblasts but increase the ability to differentiate into adipocytes, which causes bone loss, resulting in osteoporosis. For example, miR-199a-5p targeting TET2 [44], miR-218-5p targeting COL1A1 [45], and miR-291a-3p targeting DKK1 [46] promote osteogenic differentiation of hBMSCs and promote bone formation to prevent bone loss, while miR-23a-3p targeting PGC-1α [47], miR-31-5p targeting Frizzled-3 [48], and miR-128 targeting SIRT6 [49] inhibit osteoblast differentiation. Some miRNAs are involved in positive or negative regulation of hBMSC adipogenic differentiation. miR-7b-5p targeting IRS2 [50] suppresses adipogenic differentiation of hBMSCs, while miR-214-5p targeting COL4A1 [51] may promote adipogenic differentiation of hBMSCs. In the present study, miR-431-5p was proven to promote osteogenic differentiation and inhibit adipogenic differentiation of hBMSCs by targeting ROCK1, indicating the potential role of miR-431-5p in controlling the osteoblastic and adipogenic fate. Furthermore, reduced osteoblastic differentiation and enhanced adipogenic differentiation caused by hsa_circ_0006859 overexpression was partially reversed by ROCK1 knockdown, indicating that hsa_circ_0006859 suppresses osteogenesis and promotes adipogenesis via sponging miR-431-5p to upregulate ROCK1.

## Conclusions

In conclusion, exosomal hsa_circ_0006859 is upregulated in osteoporosis patients. Hsa_circ_0006859 inhibits osteoblastic differentiation and promotes adipogenic differentiation of hBMSCs by sponging miR-431-5p to upregulate ROCK1. Our findings suggest a role of hsa_circ_0006859 as a novel circulating biomarker for osteoporosis, and the mechanism of hsa_circ_0006859 induces imbalance between osteogenesis and adipogenesis, representing a novel target for osteoporosis treatment.

## Supplementary Information

**Additional file 1: Table S1.** Top five up/downregulated circRNAs in the microarray.

## Data Availability

The datasets used and/or analysed during the current study are available from the corresponding author on reasonable request.

## Abbreviations

circRNAs: Circular RNAs
qRT-PCR: Quantitative real-time PCR
ARS: Alizarin red staining
hBMSCs: Human bone marrow mesenchymal stem cells
ceRNA: Competing endogenous RNA
RBPs: RNA-binding proteins
miRNA: MicroRNA
BMD: Bone mineral density
DXA: Dual energy X-ray absorptiometry
PTH: Parathyroid hormone
25(OH)D: 25-Hydroxyvitamin D
β-CTx: β-Isomerized C-terminal telopeptides
tP1NP: N-Terminal procollagen of type l collagen
α-MEM: Alpha modified eagle’s medium
OIM: Osteogenic induction medium
AIM: Adipogenic induction medium
siRNA: Small interfering RNA
si-circ: siRNA against hsa_circ_0006859
si-ROCK1: siRNA against ROCK1
si-NC: siRNA negative control siRNA
miR-431 M: miR-431-5p mimics
miR-431I: miR-431-5p inhibitor
miR-NC: miRNA negative control
OE-circ: pcDNA 3.1 vector containing hsa_circ_0006859
OE-ROCK1: pcDNA 3.1 vector containing ROCK1 without 3′-UTR
EV: Empty vector control
ALP: Alkaline phosphatase
ARS: Alizarin red staining
Wt: Wild type
Mut: Mutant
ROC: Receiver operating characteristic
