# Correction: Exosomal hsa_circ_0006859 is a potential biomarker for postmenopausal osteoporosis and enhances adipogenic versus osteogenic differentiation in human bone marrow mesenchymal stem cells by sponging miR-431-5p

**DOI:** 10.1186/s13287-022-03096-4

**Published:** 2022-07-29

**Authors:** Feng Zhi, Yi Ding, Rong Wang, Yujiao Yang, Kaiming Luo, Fei Hua

**Affiliations:** 1 Department of Neurosurgery, Third Affiliated Hospital of Soochow University, Changzhou City, 213003 Jiangsu China; 2 Department of Geriatrics, Third Affiliated Hospital of Soochow University, Changzhou City, 213003 Jiangsu China; 3 Department of Endocrinology, Third Affiliated Hospital of Soochow University, Changzhou City, 213003 Jiangsu China

## Correction: Stem Cell Res Ther 12, 157 (2021) 10.1186/s13287-021-02214-y

Following publication of the original article [1], the authors identified that there are image overlap in the Alizarin red staining experiment in Fig. 6G EV and Fig. 6C miR-NC, Fig. 6C miR-431M, Fig. 6I si-ROCK1+EV and Fig. 6G si-ROCK1 during figure assembling. The corrected image of the Alizarin red staining experiment has been updated in Fig. 6. These errors do not affect the discussion or conclusions in the article. We, the authors regret making this mistake.

**Fig. 6:**
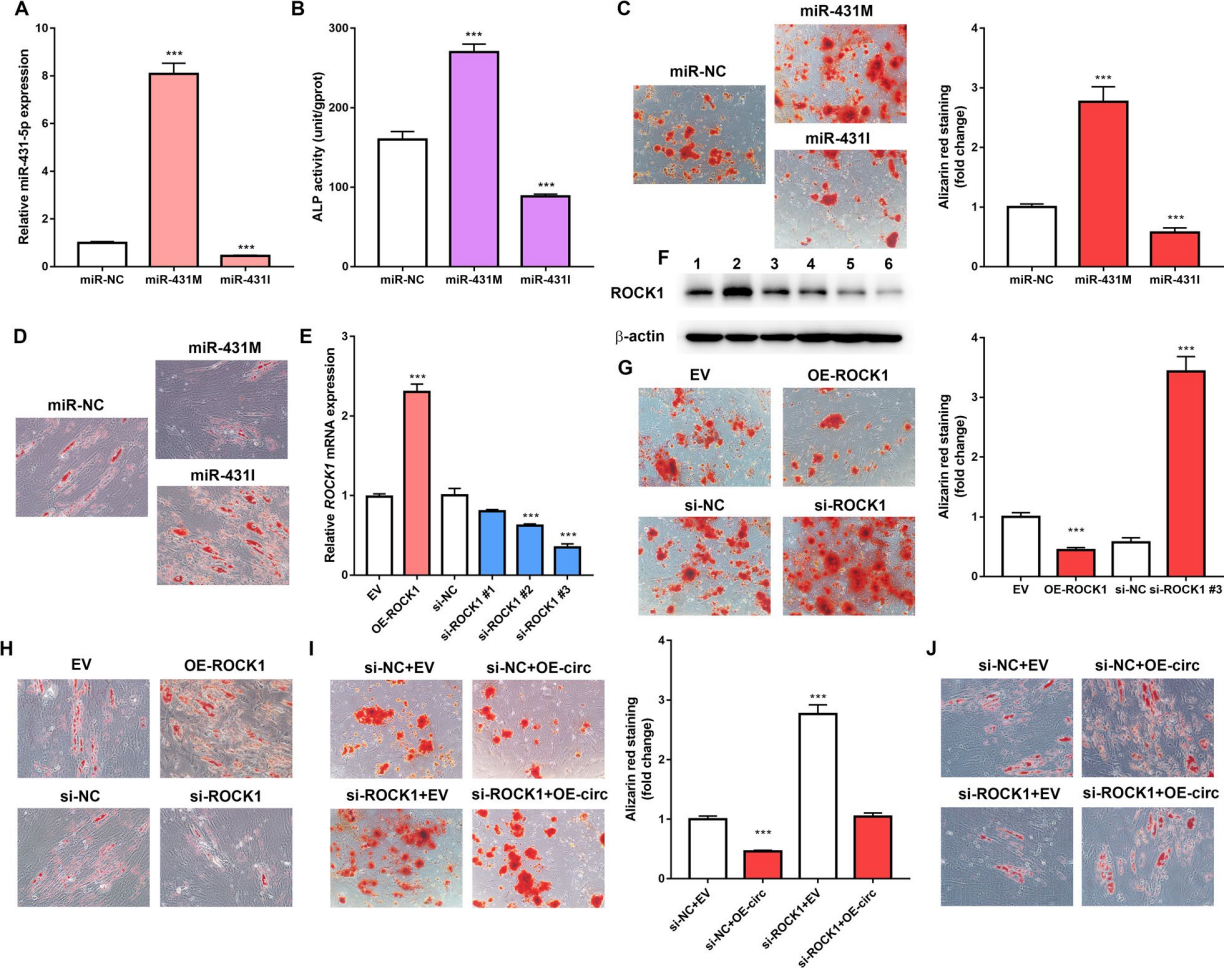
hsa_circ_0006859 suppresses osteogenesis and promotes adipogenesis by sponging miR-431-5p to upregulate ROCK1. **a** Relative miR-431-5p expression in hBMSCs after transfection with miR-431 M or miR-431I. **b** ALP activity in hBMSCs after transfection with miR-431 M or miR-431I. **c** Alizarin red staining was used to detect the mineralization ability of hBMSCs after transfection with miR-431 M or miR-431I. **d** Oil Red O staining of hBMSCs after transfection with miR-431 M or miR-431I. **e** Relative ROCK1 mRNA expression in hBMSCs after transfection with OE-ROCK1, si-ROCK1 #1, si-ROCK1 #2, or si-ROCK1 #3. **f** Western blot analysis of ROCK1 expression in hBMSCs after transfection with OE-ROCK1, si-ROCK1 #1, si-ROCK1 #2, or si-ROCK1 #3. **g** Alizarin red staining of hBMSCs after transfection with OE-ROCK1 or si-ROCK1. **h** Oil Red O staining of hBMSCs after transfection with OE-ROCK1 or si-ROCK1. **i** Alizarin red staining of hBMSCs after transfection with si-NC+EV, si-NC+OE-circ, si-ROCK1+EV, or si-ROCK1+OE-circ. **j** Oil Red O staining of hBMSCs after transfection with si-NC+EV, si-NC+OE-circ, si-ROCK1+EV, or si-ROCK1+OE-circ
