# Supplementary material for: Exosomal hsa_circ_0006859 is a potential biomarker for postmenopausal osteoporosis and enhances adipogenic versus osteogenic differentiation in human bone marrow mesenchymal stem cells by sponging miR-431-5p
Source: Stem Cell Res Ther. 2021 Mar 1;12:157. doi: 10.1186/s13287-021-02214-y (PMC7923524; doi:10.1186/s13287-021-02214-y)
Supplement: Supplementary file 1 — Additional file 1: Table S1. Top five up/downregulated circRNAs in the microarray. [file 13287_2021_2214_MOESM1_ESM.docx]

**Supplementary Online Content**

**Exosomal hsa_circ_0006859 is a potential biomarker for postmenopausal osteoporosis and controls the balance between osteogenesis and adipogenesis in human bone marrow mesenchymal stem cells by sponging miR-431-5p**

Supplementary Table 1. Top five up/downregulated circRNAs in the microarray.

| CircRNA ID | Fold change | *P*‐value | Genomic location |
| --- | --- | --- | --- |
| Upregulated |  |  |  |
| hsa_circ_0047341 | 44.00 | 0.018 | chr18 |
| hsa_circ_0006859 | 32.00 | 0.028 | chr5 |
| hsa_circ_0114134 | 30.51 | 0.0055 | chr1 |
| hsa_circ_0047337 | 25.32 | 0.02 | chr18 |
| hsa_circ_0115890 | 20.51 | 0.015 | chr21 |
| Downregulated |  |  |  |
| hsa_circ_0062454 | 0.100 | 0.025 | chr22 |
| hsa_circ_0065129 | 0.110 | 0.0029 | chr3 |
| hsa_circ_0001445 | 0.120 | 0.012 | chr4 |
| hsa_circ_0110708 | 0.120 | 0.0016 | chr1 |
| hsa_circ_0103130 | 0.120 | 0.0026 | chr15 |
